# Supplementary material for: Targeting monocytic Occludin impairs transendothelial migration and HIV neuroinvasion
Source: EMBO Rep. 2024 Jul 22;25(8):10. doi: 10.1038/s44319-024-00190-x (PMC11315906; doi:10.1038/s44319-024-00190-x)
Supplement: Supplementary file 13 — Expanded View Figures [file 44319_2024_190_MOESM13_ESM.pdf]

Expanded View Figures

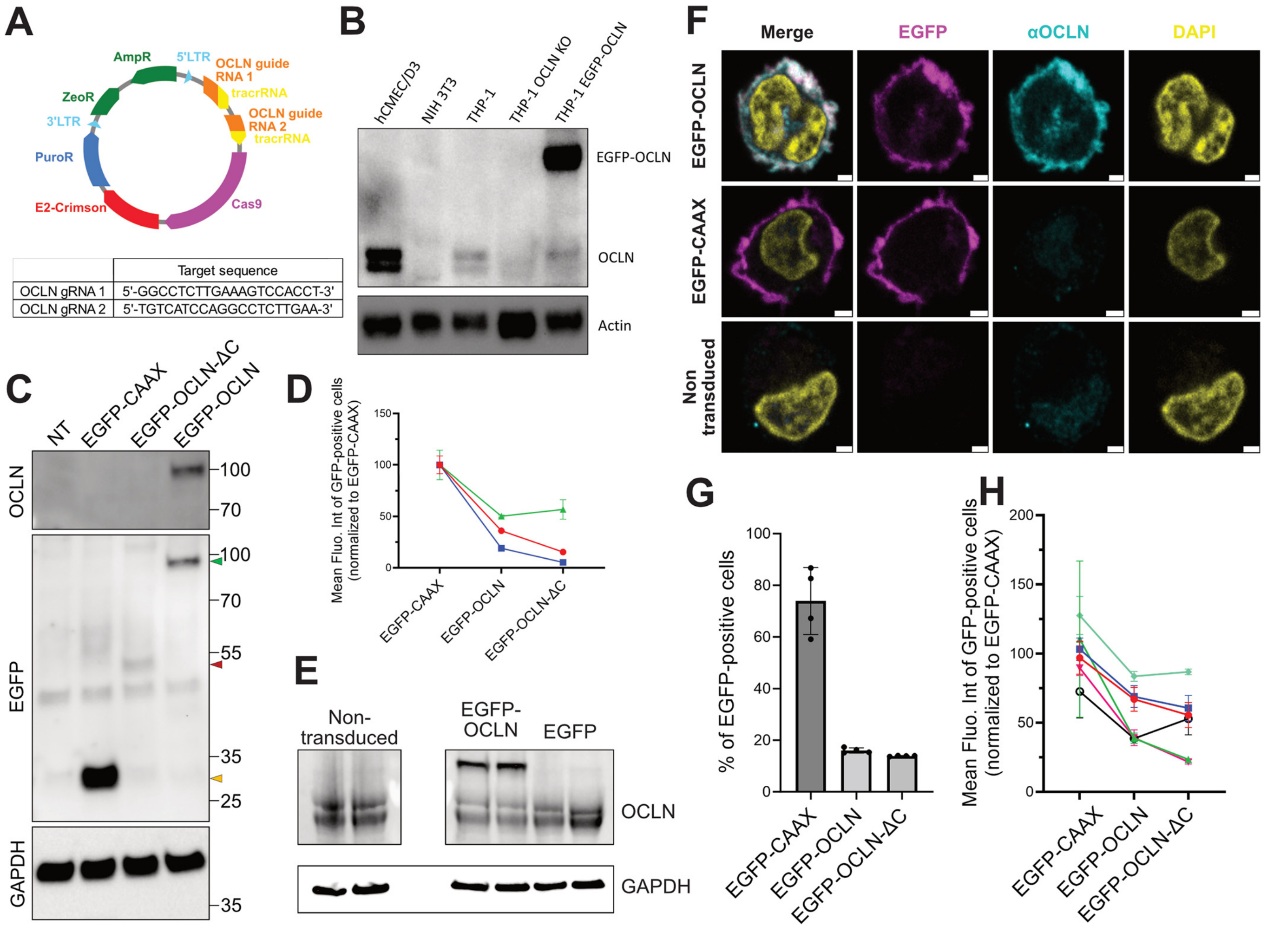

Figure EV1. Characterization of monocytic Occludin expression.

(**A**) Schematic representation of a CRISPR/Cas9 lentiviral construct used for the generation of OCLN KO THP-1 cells with indicated target sequences. (**B**) Western Blot analysis of OCLN expression in indicated cell lines. Actin is used as a loading control. The hCMEC/D3 and THP-1 cells express OCLN (two bands), the THP-1 OCLN KO cells do not show OCLN, although non-specific bands of lower size and very weak intensity can be observed. As controls, NIH 3T3 do not express OCLN and THP-1 WT transduced with EGFP-OCLN express both endogenous and overexpressed OCLN. (**C**) Western Blot analysis of OCLN expression in THP-1 OCLN KO cells. GAPDH is used as a loading control. EGFP-OCLN can be seen with both anti-Occludin and anti-GFP antibody (green arrow at EGFP). EGFP-OCLN-ΔC and EGFP-CAAX can be seen with anti-GFP antibody only (red and yellow arrows, respectively). (**D**) Mean fluorescence intensity of EGFP-CAAX, EGFP-OCLN, or EGFP-OCLN-ΔC expressed by THP-1 OCLN KO cells measured by flow cytometry. The data were normalized to EGFP-CAAX obtained from $n = 3$ individual experiments. Each symbol's color corresponds to an individual experiment. (**E**) Western Blot showing endogenous OCLN expression in human primary monocytes and exogenous expression of EGFP-OCLN in the same donors (2 donors). GAPDH is used as a loading control. (**F**) Immunofluorescence images of primary monocytes transduced with EGFP-OCLN, EGFP-CAAX, or non-transduced, fixed and stained with anti-OCLN antibody (cyan) and DAPI (yellow). EGFP is in magenta. Scale bar: 2 µm. (**G**) Efficiency of transduction of primary monocytes from 2 donors performed in two technical replicates measured by flow cytometry. (**H**) Mean fluorescence intensity of EGFP-CAAX, EGFP-OCLN or EGFP-OCLN-ΔC expressed by primary monocytes. The data were obtained from $n = 6$ monocyte donors normalized to EGFP-CAAX of each experiment. Each symbol's color corresponds to an individual monocyte donor. In (**D**, **G**, **H**), the data are presented as mean ± SEM. Source data are available online for this figure.

 © The Author(s)

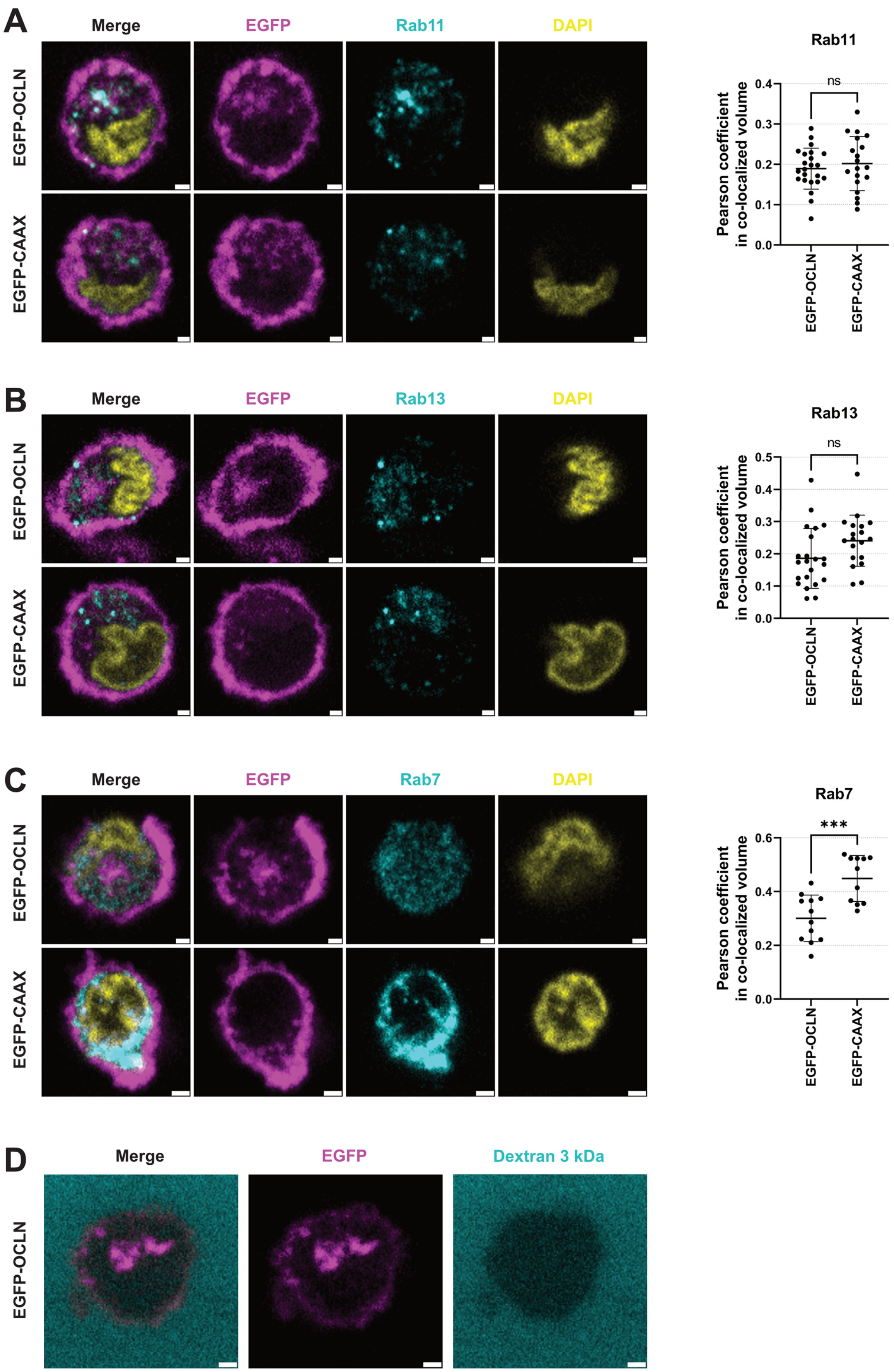
A
Merge
EGFP
Rab11
DAPI
EGFP-OCLN
EGFP-CAAX
Rab11
Pearson coefficient in co-localized volume
ns
B
Merge
EGFP
Rab13
DAPI
EGFP-OCLN
EGFP-CAAX
Rab13
ns
C
Merge
EGFP
Rab7
DAPI
EGFP-OCLN
EGFP-CAAX
Rab7
***
D
Merge
EGFP
Dextran 3 kDa
EGFP-OCLN

Figure EV2. Characterization of the OCLN-containing compartment.

(**A–C**) Immunofluorescence image of primary monocytes expressing either EGFP-OCLN or EGFP-CAAX (magenta) attached to hCMEC/D3 cells and stained with DAPI (yellow) and antibodies (cyan) against Rab11 (**A**), Rab13 (**B**), or Rab7 (**C**). Scale bar: 1 μm, except in C for EGFP-CAAX: 2 μm. (**D**) Primary monocyte expressing EGFP-OCLN (magenta) attached to hCMEC/D3 monolayer were incubated with 3 kDa fluorescent Dextran (cyan) and immediately imaged using confocal microscopy. The snapshots highlight that Dextran does not access the OCLN-containing compartment. Scale bar: 1 μm. Data information: In (**A–C**), data are presented as mean ± SEM. Two-tailed Student's t-test *p* value < 0.001 (***) or non-significant (ns). Source data are available online for this figure.

 © The Author(s)

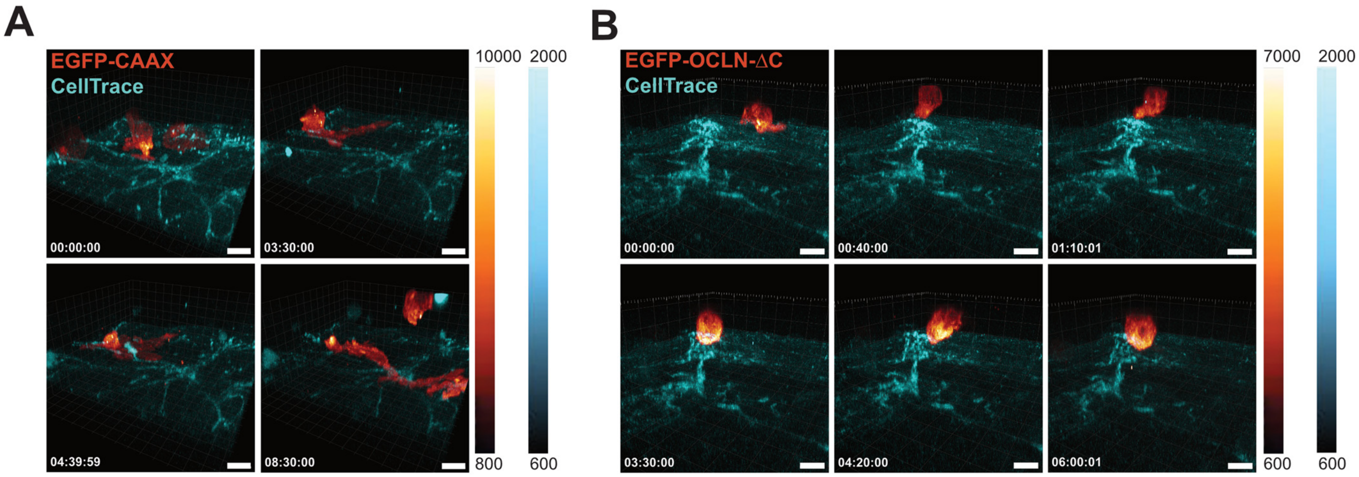

Figure EV3. Monocytic OCLN transiently polarizes at monocyte-endothelial interaction sites during transmigration.

(**A**, **B**) 3D time-lapse spinning disk confocal microscopy of transmigrating monocytes as in Fig. 1E, F. (**A**) Imaging of a primary monocyte transduced with EGFP-CAAX on hCMEC/D3 monolayer. Images were taken every 10 min. Scale bar: 10 µm. Full video can be found in Movie EV3. (**B**) Imaging of a primary monocyte transduced with EGFP-OCLN-ΔC on hCMEC/D3 monolayer. Images were taken every 10 min. Scale bar: 10 µm. Full video can be found in Movie EV4. Source data are available online for this figure.

© The Author(s)

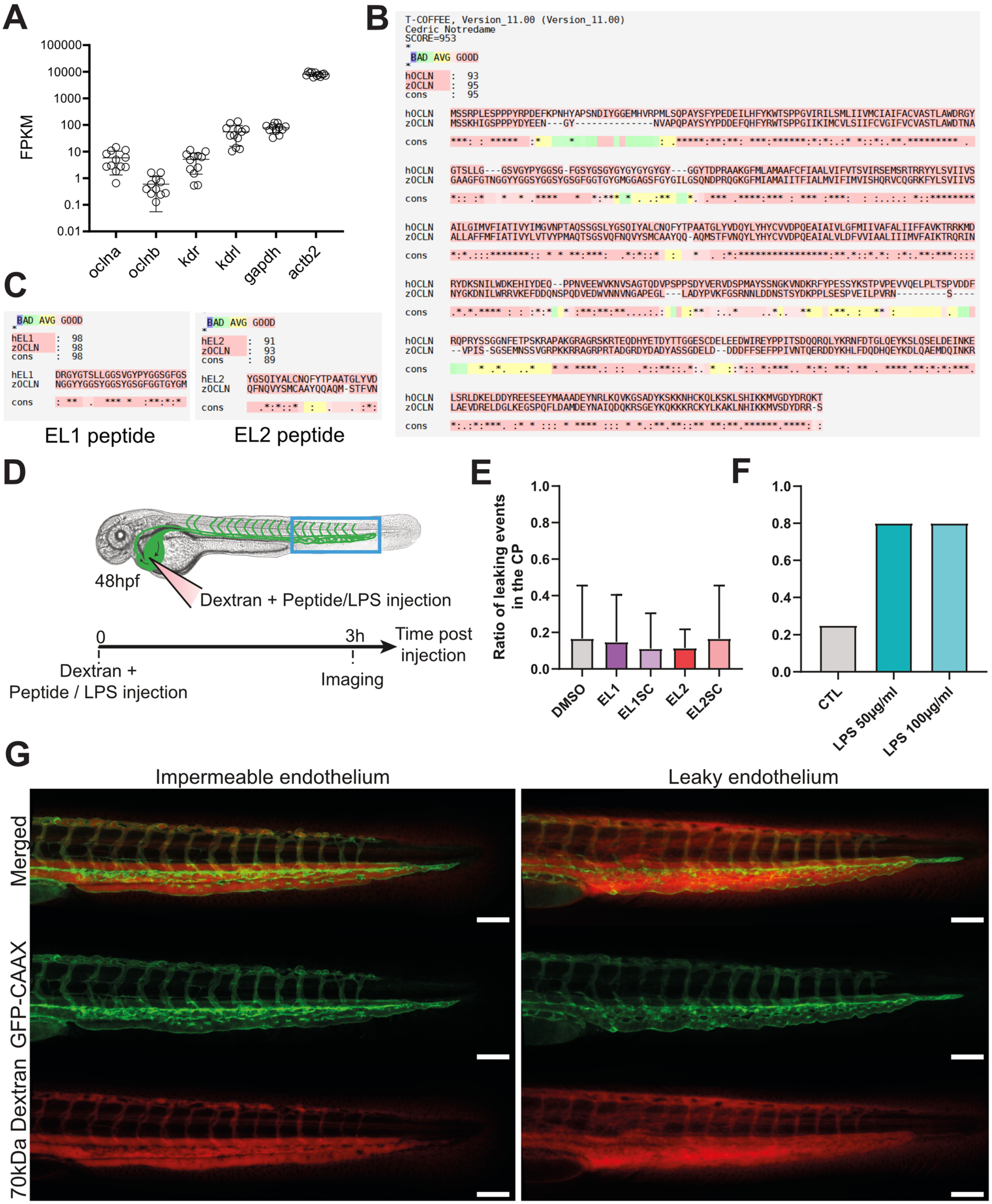
A
FPKM
100000
10000
1000
100
10
1
0.1
0.01
oclna
oclnb
kdr
kdrl
gapdh
actb2
B
T-COFFEE, Version_11.00 (Version_11.00)
Cedric Notredame
SCORE=953
BAD AVG GOOD
hOCLN : 93
zOCLN : 95
cons : 95
C
BAD AVG GOOD
hEL1 : 98
zOCLN : 98
cons : 98
hEL2 : 91
zOCLN : 93
cons : 89
EL1 peptide
EL2 peptide
D
48hpf
Dextran + Peptide/LPS injection
0
3h
Time post injection
Dextran + Peptide / LPS injection
Imaging
E
Ratio of leaking events in the CP
1.0
0.8
0.6
0.4
0.2
0.0
DMSO
EL1
EL1SC
EL2
EL2SC
F
1.0
0.8
0.6
0.4
0.2
0.0
CTL
LPS 50µg/ml
LPS 100µg/ml
G
Impermeable endothelium
Leaky endothelium
Merged
GFP-CAAX
70kDa Dextran

 © The Author(s)

Figure EV4. Characterization of the zebrafish embryo model.

(**A**) OCLN-expressing RNA for *oclna* and *oclnb* paralogues assessed in the endothelium of Tg(fli:egfp) zebrafish embryos from (Bonkhofer et al, 2019). Kdr, kdrl, *gapdh*, and *actb2* genes were used as housekeeping genes. (**B**) Comparative analysis of the human OCLN (hOCLN) and *danio rerio* (zebrafish) OCLN (zOCLN) amino acid sequences and consensus sequence using T-Coffee (see Methods for details). Red underlining of amino acids indicates good sequence similarity. (**C**) Comparative analysis of the EL1- and EL2-derived sequences of hOCLN with zOCLN. Red underlining of amino acids indicates good sequence similarity. (**D**) Schematic of the experimental procedure associated with the zebrafish model to test the effect of EL1, EL2 and their scramble peptides on endothelial permeability. Treatment with LPS is used as control for permeability inducing agents. (**E**) Bar graph of the ratio of leaky endothelium at 3 h post dextran and peptide injection. The experiment was carried out three independent times ($n_{DMSO} = 39$; $n_{EL1} = 25$; $n_{scEL1} = 26$; $n_{EL2} = 24$; $n_{scEL2} = 23$). (**F**) Bar graph of the ratio of leaky endothelium at 3 h post dextran and peptide injection. The experiment was carried out one time. ($n_{Control} = 12$; $n_{LPS\text{-}50 \mu g/mL} = 15$; $n_{LPS\text{-}100 \mu g/mL} = 15$). (**G**) Representative images of zebrafish embryos with an impermeable or leaky endothelium in the tail at 3 h post injection of PBS or LPS, respectively. Scale bar: 100 µm. Data information: In (**A**, **D**, **E**), data are presented as mean ± SD.

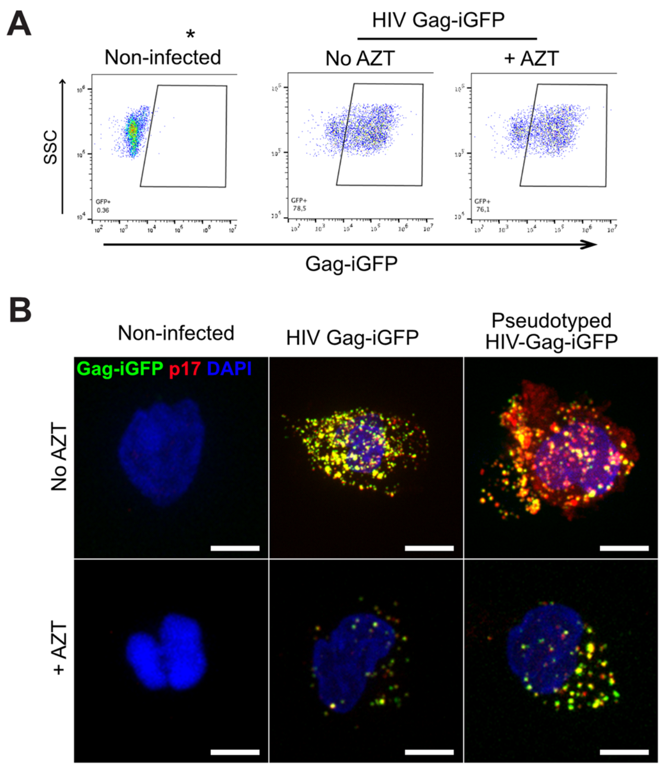

Figure EV5. Characterization of primary monocyte infection by HIV-1 and transmigration.

(**A**) Primary monocytes were non-infected or infected with HIV-1 (NLAD8) at MOI 1 for 48 h in the presence or absence of 10 μM AZT. The dot plots show the percentage of Gag-iGFP-expressing cells as a function of the side scatter (SSC) measurement as determined by flow cytometry. The data highlights that despite AZT treatment, monocytes are positive for Gag-iGFP as they carry fluorescent particles. (**B**) Primary monocytes were non-infected, infected with HIV-1 Gag-iGFP, or HIV-1 Gag-iGFP pseudotyped with VSV-G, at MOI 1 for 48 h in the presence or absence of AZT. Cells were fixed and stained for Gag p17 (red) and DAPI (blue) and Gag-iGFP is shown in green. Scale bar: 5 μm.

 © The Author(s)
